# Subcutaneous infliximab in inflammatory bowel disease: bridging the gap between theory and practice

**DOI:** 10.1093/crocol/otag010

**Published:** 2026-02-05

**Authors:** Lucine Vuitton, Mathurin Fumery, Lucile Foulley, Caroline Habauzit, Salim Benkhalifa, Anthony Buisson

**Affiliations:** Department of Gastroenterology, University Hospital of Besançon, UMR 1098 Right Inserm, Université de Franche-Comté, Besançon, France; Department of Gastroenterology, Centre Hospitalier Universitaire de Amiens, and Peritox, Université de Picardie Jules Verne, Amiens, France; Celltrion Healthcare France, Issy les Moulineaux, France; Celltrion Healthcare France, Issy les Moulineaux, France; Celltrion Healthcare France, Issy les Moulineaux, France; Inserm, 3iHP, CHU Clermont-Ferrand, Service d’Hépato-Gastroentérologie, Université Clermont Auvergne, Clermont-Ferrand, France; Inserm U1071, M2iSH, USC-INRA 2018, Université Clermont Auvergne, Clermont-Ferrand, France

**Keywords:** subcutaneous, infliximab, inflammatory bowel disease (IBD), Crohn’s disease, ulcerative colitis

## Abstract

**Background:**

At the end of the past century, infliximab (IFX), the first-in-class biological therapy approved in inflammatory bowel disease (IBD), dramatically modified the therapeutic armamentarium. The recent development of subcutaneous (SC) formulations of IFX offers a promising alternative, with the potential to improve patient convenience, adherence, and overall outcomes. This review explores the clinical evidence supporting the initiation of SC IFX and the transition from intravenous (IV) to SC IFX.

**Methods:**

Comprehensive review using MEDLINE (source PUBMED).

**Results:**

Comparative studies have shown that SC IFX is associated with higher IFX serum concentration levels than IV, fewer neutralizing antibodies and similar levels of remission. Real-world studies have confirmed that switching from IV to SC IFX 120 mg is well accepted with a low risk of relapse. The ease of at-home administration has been associated with improved patient satisfaction and reduced healthcare burdens. The adoption of SC IFX could profoundly change the therapeutic landscape, offering a more patient-centered approach to long-term disease control but some questions remain, particularly about the place of IFX in certain populations.

**Conclusion:**

In this article, we reviewed the known and unknown data on SC IFX to provide a comprehensive summary and assist IBD physicians in integrating this knowledge into everyday clinical practice.

## Graphical Abstract

**Graphical Abstract:**
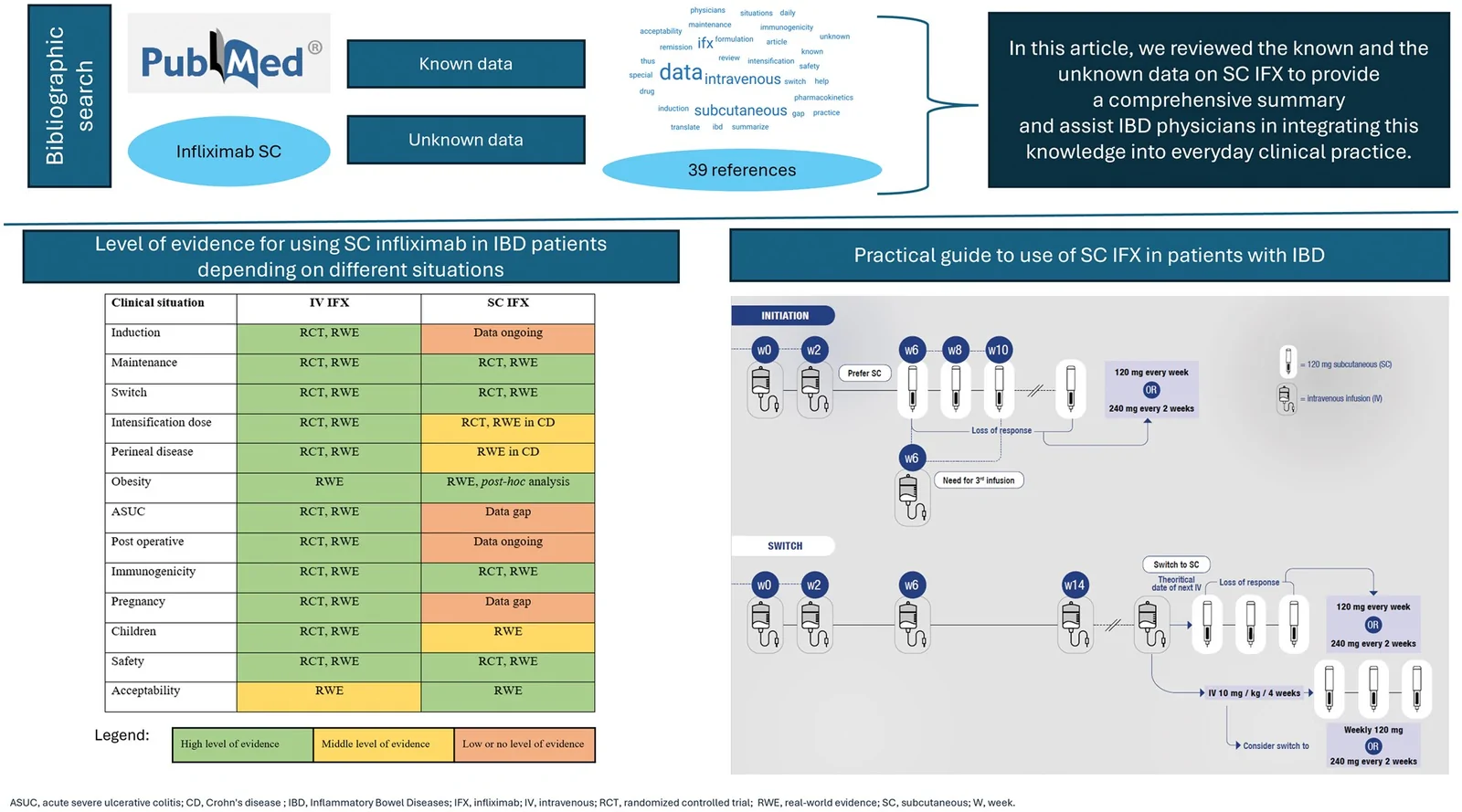

## Introduction

**Figure 1:**
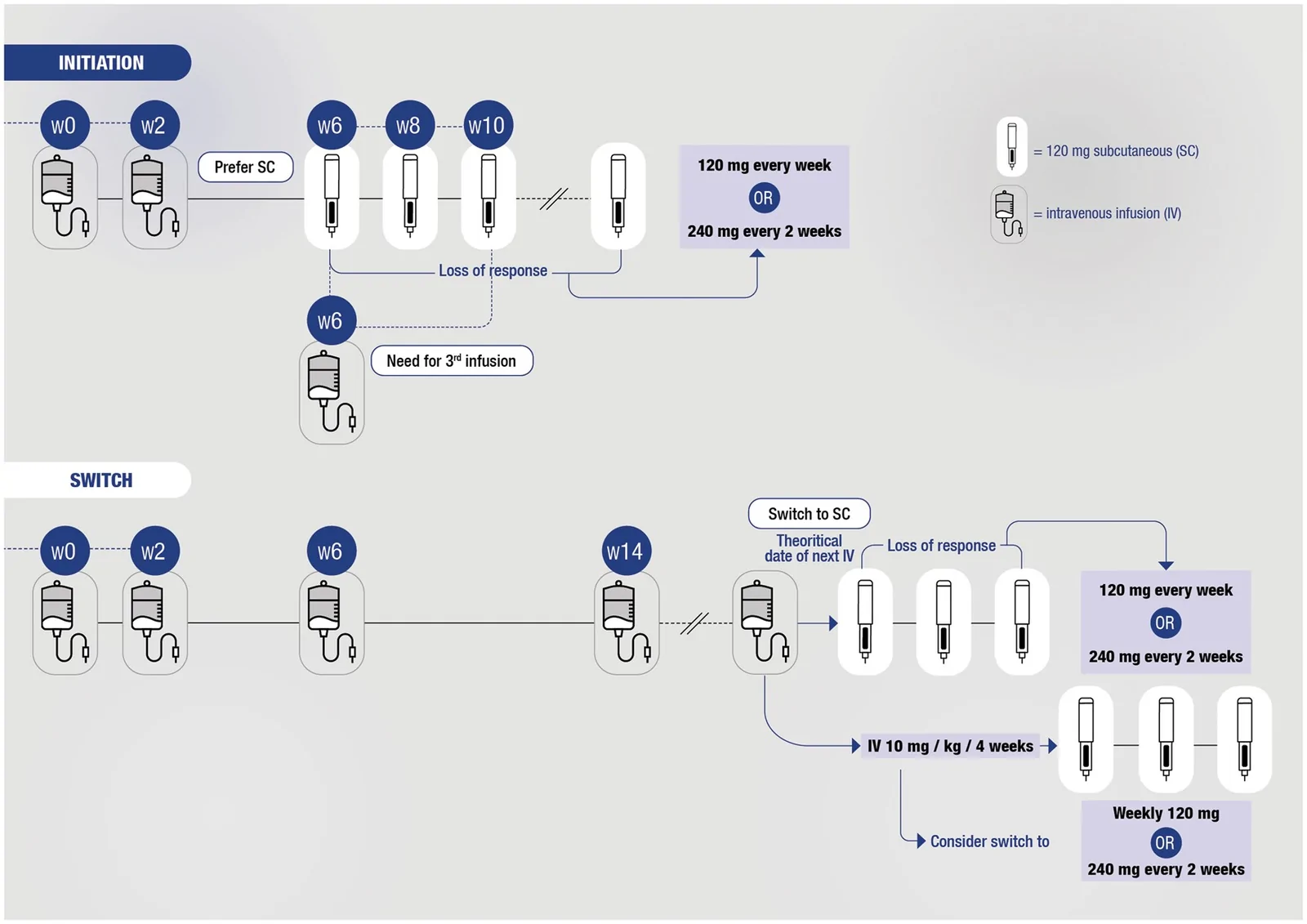
Practical guide to use of subcutaneous (SC) infliximab (IFX) in patients with inflammatory bowel disease (IBD).

In the 90’s, infliximab (IFX) was the first-in-class biological therapy approved for inflammatory bowel disease (IBD) (first approval for Crohn’s disease [CD] in 1998) and dramatically modified the therapeutic armamentarium to manage patients with IBD. Intravenous (IV) IFX demonstrated its efficacy to induce and maintain remission in both CD^1^ and Ulcerative colitis (UC),^2^ including special situations such as perianal disease^3^ and acute severe ulcerative colitis (ASUC).^4^ Combination therapy with thiopurines demonstrated higher efficacy than IV IFX alone and is now recommended in most situations.^5^^,^^6^ Moreover, the pharmacokinetic profile of IV IFX has been widely deciphered highlighting high potential for immunogenicity.^7^ Recently, a subcutaneous (SC) formulation of IFX has been developed and approved by Europe Medicines Agency (EMA) in 2021 (Figure 1) owing to its noninferiority compared to IV IFX based on pharmacokinetic data^8^ and more recently in United States by Food and Drug Administration (FDA) based on 2 randomized phase 3 trials. SC IFX is not considered as a biosimilar by regulatory agencies and the nonconsensual term of biobetter^9^ is sometimes evocated to refer to SC IFX due to expected better acceptability and convenience as well as potential improved pharmacokinetic profile.

In this article, we will review data on induction and maintenance of remission, switch from IV to SC formulation, drug intensification, pharmacokinetics and immunogenicity, special situations, data gap, safety, and acceptability of SC IFX. Thus, we will summarize the known and the unknown data on SC IFX to help IBD physicians to translate it into daily practice.

## Literature search

MEDLINE (source PUBMED, 1966 to May, 2025) was employed using a combination of keywords, namely: (“infliximab” OR “CT-P13”) AND “subcutaneous” AND (“Crohn’s disease” OR “Ulcerative colitis” OR “inflammatory bowel disease”). The literature search identified 198 articles. The authors graded the abstract of every trial identified by the search to determine eligibility. If these criteria remained unclear from the abstract, the full article was retrieved for clarification. The relevant articles are listed in Table S1.

## Clinical situations with available data on SC IFX

The level of evidence for both IV and SC IFX is summarized in Table 1 for each following situation.

**Table 1:** Level of evidence for using SC infliximab in IBD patients depending on different situations.

Clinical situation	IV IFX	SC IFX
**Induction**	RCT, RWE	Data ongoing
**Maintenance**	RCT, RWE	RCT, RWE
**Switch**	RCT, RWE	RCT, RWE
**Intensification dose**	RCT, RWE	RCT, RWE in CD
**Perineal disease**	RCT, RWE	RWE in CD
**Obesity**	RWE	RWE, post hoc analysis
**ASUC**	RCT, RWE	Data gap
**Postoperative**	RCT, RWE	Data ongoing
**Immunogenicity**	RCT, RWE	RCT, RWE
**Pregnancy**	RCT, RWE	Data gap
**Children**	RCT, RWE	RWE
**Safety**	RCT, RWE	RCT, RWE
**Acceptability**	RWE	RWE

Abbreviations: ASUC, acute severe ulcerative colitis; CD, Crohn’s disease; IFX, infliximab; IV, intravenous; RCT, randomized controlled trial; RWE, real world evidence; SC, subcutaneous.

We considered high level of evidence when: (1) the protocol is adapted to best answer the question posed; (2) the study is carried out without major bias; (3) the statistical analysis is adapted to the objectives; (4) the power is sufficient. Middle level of evidence when (1) the protocol is adapted to best answer the question posed; (2) clearly insufficient power (insufficient numbers or insufficient a posteriori power); (3)and/or minor anomalies. Low or no level of evidence when (1) other type of study; (2) no study.

### Induction and maintenance therapy

Two randomized controlled trials (RCTs) evaluated SC IFX maintenance in patients with active CD and UC after IV induction. In the pivotal study reported by Schreiber and colleagues,^8^ so called the 1.6 study, anti-TNF naive patients with active UC (*n* = 66) or CD (*n* = 65) received 2 IV infusions of IFX at Week 0 and Week 2 and were subsequently randomized (1:1) to receive SC IFX every other week (eow) from Week 6 to Week 54, or IV IFX every 8 weeks from Week 6 to Week 22. At Week 30, all patients receiving IV IFX switched to SC IFX eow until W54. The primary endpoint, which was noninferiority of SC IFX to IV IFX for observed predose IFX concentration at Week 22 (Ctrough, w22), was met. Exploratory efficacy endpoints showed that clinical remission at Week 30 was achieved in 60.5% and 53.8% of the patients with UC in the SC IFX and IV arms, respectively while clinical remission was obtained in 64.3% and 56% of the patients with CD in the SC IFX and IV arms, respectively. Open label design and small sample size of this pivotal trial precludes firm conclusions on the superiority of one route versus another.

A *post hoc* analysis^10^ of this trial investigated clinical outcomes in patients treated with SC or IV IFX, among the subgroup of patients who did not achieve clinical response at Week 6 after IV induction. Overall, 17 and 13 patients were ­nonresponders after IV induction in the SC and IV arms, respectively. Exploratory and nonrandomized comparison of these small sample size groups found numerically a 2-fold higher rate of clinical remission at Week 30 in patients receiving SC IFX from Week 6 compared to those continuing IV infusions (58.8% vs. 30.8%, respectively; *P* = 0.159). It was suggesting potential benefits of SC IFX in this situation. However, given the small size population and potential attrition bias, we cannot draw any firm conclusion, and dedicated investigations are warranted to address this question.

The LIBERTY^11^ randomized, placebo-controlled trials aimed to demonstrate the superiority of SC IFX over placebo as maintenance therapy in patients with CD and UC. Patients with moderately to severely active CD (*n* = 396) and UC (*n* = 548) received 3 open-label IV IFX perfusions at Weeks 0, 2, and 6. At Week 10, clinical responders were randomized (2:1) to receive either SC IFX 120 mg eow or placebo until Week 54. In CD, SC IFX was more effective than placebo to achieve clinical remission (62.3% vs. 32.1%; *P* < .0001) and endoscopic response (51.1% vs. 17.9%; *P* < .0001) at Week 54. In the same way, SC IFX was more effective than placebo to obtain clinical remission at week 54 in patients with UC (43.2% vs. 20.8%; *P* < .0001). In addition, all secondary endpoints including biomarkers and endoscopy data, were met in both CD and UC.

Real-world data are currently scarce regarding the use of SC IFX after IV induction. One study investigated the 1-year real-life effectiveness and durability of SC IFX in South Korea.^12^ Patients with UC and CD received 2 IFX IV infusions at Weeks 0 and 2, and subsequent SC IFX eow from Week 6. A total of 192 patients (53 UC and 139 CD) were enrolled. At Week 26, clinical remission rates were 54.5% and 92.1% according to partial Mayo score and CDAI, in UC and CD, respectively. Week 26 drug survival rate was over 98% in both UC and CD patients.

### Switch from IV to SC therapy

The switch from IV to SC IFX refers to patients currently in remission while on IV maintenance regimen and accepting to move to SC IFX. This situation has to be distinguished from the SC maintenance regime starting immediately after the 2 or 3 IV infusions described above. After the publication of the pivotal trial by Schreiber and colleagues^8^ including exploratory data suggesting a potential improvement of clinical and biological parameters, real-world data accumulated on such a switch.^13–17^ While this route of administration may improve patients’ ­acceptability,^18^ 75% of the patients accepted the switch in the 2 real-world evidence cohorts reporting the total number of ­eligible patients at baseline.^14–17^ Among IBD patients in clinical remission who accepted the switch, the drug retention was very high after switching from IV to SC IFX in real-world cohorts as it consistently overpassed 90%.^14–17^^,^^19^ However, drug retention is a minimalist endpoint that only partially reflects patient and physicians’ expectations. Avoiding clinical relapses appears as a more relevant target for IBD patients in clinical remission accepting the switch. As real-world evidence data are mainly not comparative, this risk of relapse should be indirectly compared to the usual risk of secondary loss of response with IV maintenance therapy (10%-20% patient-years).^20^ In the REMSWITCH study, a multicenter observational trial enrolling 133 patients, the rate of relapse after switching from IV to SC IFX (120 mg eow) was low in patients treated with regular IV regimen, that is.5 mg/kg/8 weeks, (13.7% at 18 months)^13^^,^^14^ without any increased risk in those treated initially at 10 mg/kg/8 weeks (15.8% and 21.2% at 6 and 18 months, respectively).^13^^,^^14^ Patients treated with 10 mg/kg/6 weeks did not present with a higher risk of relapse after switching to SC 120 mg eow at 6 months (17.6%), but an increase of delayed relapse was observed at 18 months (37.5%).^14^ The subgroup of patients with highly intensified IV doses (10 mg/kg/4 weeks) experienced higher rates of relapse after switching to SC 120 mg eow: 66.7% at 6 months and 92.9% at 18 months.^13^^,^^14^ However, in this subgroup, SC dose escalation was effective (recapture of response in 82.1%) and similar risk of treatment discontinuation was observed as compared to other initial IV regimens. These data suggest that patients with highly intensified IV regimen could benefit from a switch to immediately intensified SC regimen (120 mg weekly or 240 mg eow).^14^ Based on the design of the REMSWITCH program and clinical relevance, the most suitable time to transition from IV to SC IFX seems to be the theoretical time of the next IV infusion.^13^ The only risk factors of relapse after the switch were persistence of inflammatory activity and initial IV regimen.^13^^,^^14^ In population pharmacokinetic simulations to explore the exposure profiles following various label and off-label IV-to-SC dosing scenarios, the authors showed switching 4 instead of 8 weeks after the last IV IFX dose may hit serum IFX steady state faster, thereby avoiding the risk of temporary underexposure. However, the clinical relevance of this theoretical risk is currently unknown.^21^

In a French retrospective study, among 27 patients who were switched back to IV IFX (after failing to switch form IV to SC IFX), only 4/27 patients permanently discontinued IFX (median follow-up of 46 weeks).^22^

### Drug intensification

Dose escalation to 240 mg every other week (eow) or 120 mg weekly (ew) offers a chance to regain response in patients with loss of response with SC IFX. In the REMSWITCH-LT study,^14^ 28 ­patients had their dose increased to either 240 mg eow or 120 mg ew after relapse. A total of 82% of patients regained response, with no significant difference between the 2 dosing regimens. In the LIBERTY trial,^11^ dose intensification to SC IFX 240 mg was permitted for patients who initially responded but subsequently lost response after Week 22. At Week 54, 32% (*n* = 7/22) of patients with CD and 30% (*n* = 13/43) of patients with UC achieved steroid-free clinical remission without any safety concerns.^11^

Since patients transitioning from optimized IV IFX to SC IFX may be at increased risk of relapse, immediate switching to an ­optimized dose (240 mg every other week or 120 mg weekly) has been proposed.^13^ In the Liverpool cohort, among the 50 patients who had an increased frequency of IV IFX at baseline (5 mg/kg every 4 or 6 weeks), there were no significant differences in treatment persistence rates, fecal calprotectin levels, or IFX concentrations between the 2 SC IFX dosing regimens at 3, 6, and 12 months. However, those on the intensified dose had significantly lower CRP levels.^16^ Finally, the AMARETTO randomized trial (ClinicalTrials.gov ID NCT06113913), which will compare the rate of steroid-free clinical remission at Week 52 after switching to either intensified IFX SC or continuing the intensified IV IFX regimen, should provide further insights into this issue.

### Pharmacokinetics and immunogenicity

The PK analysis of the CT-P13 SC 1.6 study found higher C-trough values for SC than for IV dosing. In the IV IFX arm, predose serum IFX concentrations gradually decreased up to W30 as the dosing interval increased. Conversely, serum concentrations remained ­relatively stable with minimal fluctuations in the SC IFX group.^8^ Similarly, during the maintenance phase of the LIBERTY study,^11^ predose serum concentrations in the SC IFX group increased at Week 14 and maintained a consistent level up to Week 54 (CD: 13.2-14.8 mg/mL; UC: 14.6-16.3 mg/mL).^11^ These data suggest that drug concentration thresholds used in clinical practice for IV IFX are not directly applicable to SC IFX, and this is probably related to its mode of administration. These data were confirmed in real-life studies. In the REMSWITCH study, the variation of serum levels before and after the switch was associated with the risk of relapse.^13^^,^^14^ Patients with reduced but also with stable levels of serum IFX after the switch had a higher risk of relapse than those with increased levels (58.3%, 52.6%, and 19.7% at 18 months, respectively).^13^^,^^14^ In a population PK simulation study,^23^ a linear relationship was observed between IV and SC trough concentrations at steady state. However, whether trough concentration or overall drug exposure (AUC) drives treatment response in IBD is still a matter of debate. Besides, Q2W SC dosing regimen of IFX has been selected with the purpose of generating a steady state 8 weeks drug exposure AUC that aligns closely to that achieved by Q8W 5 mg/kg IV dosing.^23^ At steady state (Weeks 22-30), in the 1.6 trial, it was observed that mean serum concentrations were stable and minimally undulating in the CT-P13 SC arm, but mean AUC exposure through the 8-week interval was only slightly higher for CT-P13 SC versus CT-P13 IV.^8^

Roblin et al. reported that the therapeutic targets regarding the serum level of SC IFX are higher than those observed for IV IFX, with SC IFX thresholds varying from 12 μg/L (sustained clinical remission) to 20 μg/L (deep remission) (AUC = 0.84) depending on the depth of the remission.^24^ In another Spanish multicenter observational study, the suggested optimal cutoff concentration for clinical and biochemical remission was 12.2 μg/mL (AUC 0.62).^15^ Finally, the pharmacological study from Roblin and colleagues recently demonstrated that IFX drug levels remained stable throughout the 14-day SC treatment cycle.^25^ It means that SC maintenance therapy leads to very low variation in serum concentration levels. From these data, a pragmatical message for clinical practice could be that therapeutic drug monitoring of IFX can be performed at any time between 2 SC injections.

In the 1.6 study, the rate of patients with ADA was not different between the 2 groups (drug-tolerant assay).^8^ Additionally, there was no increase in immunogenicity after patients switched from IV to SC IFX. However, a significantly lower proportion of patients receiving SC IFX developed neutralizing antibodies. Interestingly, there was a trend for lower rates of ADA and neutralizing antibodies postswitch compared to preswitch. In the LIBERTY trials, the proportion of patients with positive ADA in the SC maintenance phase was 65% and 64% for CD and UC, respectively.^11^ However, a drug-tolerant assay was used, and neutralizing antibodies were not assessed. These findings were in line with those from real-world studies. In the REMSWITCH study, none of the 133 patients newly developed ADAs over 24 weeks of treatment (drug-sensitive assay).^13^^,^^14^ In the Liverpool study, 14 patients (8%) newly developed ADA after 12 months.^16^ In the multicenter Spanish study, no ADAs were observed during the 12-month follow-up after switching to IFX SC.^15^ Another real-life study had evaluated the effectiveness of SC IFX among patients unsuccessfully treated by at least 2 biologics and including 20 (62%) patients previously treated by IV IFX. Through serum IFX median values showed rapid increase and exceeded 15 μg/mL at W30. Interestingly, in the subgroup of 17 patients with antidrug antibodies (ADA) at baseline, ADA was no more observed in 10 patients whereas median ATI levels significantly declined. Among ADA-negative patients at baseline (*n* = 15), only 1 developed ADA at W30.^26^ Thus, it could be hypothesized that SC IFX could provide pharmacokinetic advantages over IV, with high and stable trough serum concentrations, leading to a lower risk of immunogenicity.

### Perianal disease

CD with perianal involvement management is challenging as perianal disease are disabling and associated with worse outcomes and frequent relapse.^1–5^ IFX has demonstrated its efficacy in phase 3 trials on perianal CD^6^^-^8 and currently is the cornerstone treatment for this phenotype.^9^^,^^10^ SC IFX has not been specifically evaluated in perianal CD in randomized trials. However, its favorable pharmacokinetic profile may be an advantage, and some data are emerging from real-world clinical practice. A large retrospective multicenter cohort study was reported by the GETAID.^27^ Patients with either active perianal CD at the initiation of IFX with subsequent SC injections (*n* = 66); or patients with inactive perianal CD on IV IFX maintenance therapy for at least 6 months and switching to SC IFX switch were included (*n* = 117). In patients with active perianal CD, clinical remission and response were observed in 44.6% and 87.7% at 6 months. Median SC IFX serum levels were >20 μg/L and only 3 patients developed ADA. In multivariable analysis, high body mass index (BMI) was the only independent predictor of remission (OR: 0.88, 95% CI: 0.77-0.99). In patients with inactive perianal CD under IV IFX, the rates of relapse-free survival were 94.3% and 87.9% at 6 and 12 months after switch. Small subgroups of patients with perianal CD were also studied from observational cohorts of switch from IV to SC IFX.^15^^-^16 In 2022, the Liverpool cohort reported data on the effectiveness of SC IFX in 181 patients with IBD and observed 2/25 perianal relapses.^16^ Nine patients with inactive perianal CD were included in the study by Huguet et al., and no worsening of perianal disease was observed.^15^ In the REMSWITCH study, among the 133 patients enrolled, 40 patients with CD had a prior history of anoperineal lesions including 3 with active lesions. During the follow-up, only 1 patient (2.5%) treated with IFX 10 mg/kg/6 weeks before the switch, presented with perianal relapse.^13^

### Obesity

In some clinical situations, IBD physicians could be reluctant to use SC rather than IV IFX. A first particular situation is patients suffering from obesity. Elevated BMI was associated with decreased drug bioavailability^28^ and worse disease outcomes.^24^^,^^28^ However, a post hoc study of the REMSWITCH study specifically focusing on the subgroup of obese patients, did not report any increased risk of relapse in this population.^25^ As a reminder, the population eligible for switching was patients in remission on a given IV dose that had already been adapted according to the issues linked to inflammatory load and drug clearance. This may explain why the main risk factor for relapse at the time of switching to SC 120 mg every 2 weeks was the IV regimen. The situation of SC maintenance regimen after 2 or 3 initial IV infusions is a distinct situation. Although BMI is not the only parameter affecting drug clearance, it could influence drug distribution and IBD physicians should be prompted to intensify medication dose in case of insufficient response. In the GETAID cohort on perianal CD, the only factor inversely associated with 6 months remission in patients with active perianal disease at IFX initiation was BMI.^27^ In the LIBERTY trials, clinical remission was achieved in 66.2% of normal-weight versus 44.4% of obese patients. While the difference was not statistically significant, it could be due to the small sample size (*n* = 18 in the obese group).^29^ The LIBERTY study also reported significantly lower trough IFX levels in obese patients, which aligns with current knowledge of drug pharmacokinetics in this population.

### Previous anaphylaxis or immunogenicity to IV

The feasibility of SC IFX therapy in patients who had previously discontinued IV IFX due to severe immediate hypersensitivity reactions, immunogenicity, or both, has been reported in a small case series. After failure with other available treatments, 3 patients received SC IFX without IV induction, and none experienced a serious reaction to the SC IFX. Clinical response was observed in 2 of these patients, while 1 patient had to discontinue treatment due to a disabling injection site reaction.^30^ The level of evidence is however insufficient to propose SC IFX among patient with IV IFX hypersensitivity reaction in clinical practice while waiting for new data. Husman et al. evaluated the effectiveness and safety of SC IFX among 20 patients with loss of response and immunogenicity to IV IFX. No immediate hypersensitivity reactions were observed. Two patients discontinued IFX SC treatment because of delayed hypersensitivity at Week 2 and Week 4. IFX serum concentrations increased from baseline to Month 12, while anti-drug antibody levels decreased. Combined clinical and biochemical remission at Month 12 was observed in 7 of 20 patients (35%).^31^

## Clinical situations with no or almost no data on SC IFX

### Full SC IFX induction without IV infusion

Initiating IBD treatment with SC IFX without using IV induction is one of the key data gaps today, as some patients are not able to come to the hospital for IV infusions and some IBD specialists do not have access to IV rooms. PASSPORT is an ongoing study in France (ClinicalTrials.gov ID NCT06274294) that aims to compare pharmacokinetics of IV versus full SC IFX induction therapy in adult patients with IBD. The study, which is currently recruiting, aims also to assess the efficacy and the safety of this SC induction.

### Combination therapy versus monotherapy at the time of IFX initiation

The current practice is to start IV IFX in association with an immunosuppressant (IS),^5^^,^^32^ which is mainly due to the immunogenicity profile of IV, occurring most frequently within the first year of treatment. With the PK profile of SC IFX showing a stable serum IFX concentration over time, using SC IFX without IS could be an interesting option. A post hoc analysis from LIBERTY studies found no difference of efficacy or safety when SC IFX was used in monotherapy, as compared to combination therapy with an IS.^33^ A post hoc small sample size sub-study of 1.6 trial showed similar drug target exposure, neutralizing antibodies and bio-clinical outcomes in monotherapy and combotherapy patients. However, as discussed before, current evidence is insufficient to support a claim that SC IFX is less immunogenic than IV.^34^ Results from the ongoing DIRECT CD study (EUDRACT 2021-000469-33), a multicentre RCT that aims to compare the efficacy of SC IFX with or without IS to induce and maintain remission in patients with CD, are eagerly awaited.

### Pregnancy

A significant proportion of female with IBD are of childbearing age and could desire to become pregnant. Therefore, generating safety data for the mother and the future baby remains important, especially due to the high serum levels of SC IFX. However, there is currently no available data regarding exposure to SC IFX during pregnancy.

### Children

Despite evidence in adult patients, there is limited data on SC IFX in pediatric IBD as SC IFX is still not approved in this specific population.

A single-center cohort experience of an elective switching program from maintenance biosimilar IV IFX to SC IFX 120 mg eow, reported that 7 pediatric patients remained in clinical remission with no significant changes in laboratory markers and median IFX trough levels (12.3 µg/mL at baseline; 13.9 and 14.0 µg/mL at 6 and 40 weeks, respectively). No newly developed ADA was detected, and no adverse reactions or rescue therapies were recorded.^35^

In France, data from a multicentre retrospective cohort, reporting the use of SC IFX in 21 IBD patients under 18 years old, concluded to the absence of relapse at 6 months postswitch to SC IFX with stable IFX trough levels and good safety profile and acceptability (data not published yet).^36^

However, these studies included mainly adolescents with small sample size, highlighting the need for dedicated data in these young patients with IBD.

### Acute severe ulcerative colitis

Nearly a quarter of UC patients will experience ASUC in their lifetime, including 30% who will fail first-line corticosteroid therapy that will require rescue therapy with IFX, ciclosporin, or ­colectomy. IFX and ciclosporin are both effective second-line therapies, as shown in a RCT.^4^ But at this time, there is no evidence evaluating efficacy of SC IFX in this specific situation.

### Other situations

Only scarce data exists on extra-intestinal manifestations. In the French PEREM prospective cohort, the rate of extraintestinal manifestations was low: the most frequent was rheumatologic. Among 28 patients with axial or peripheral arthralgia, 19 had no more symptom at W48; 1 patient had uveitis at baseline, which recovered during follow-up. Dedicated and prospective cohorts are necessary to conclude on that point. There is currently no data on SC IFX in the postoperative setting, however, the POMEROL study (NCT05072782), exploring postoperative Rutgeerts i2-recurrence treated with SC IFX, is ongoing.

## Safety

In the pivotal study reported by Schreiber and colleagues, the incidence of drug-related treatment-emergent adverse events (TEAEs) (58% and 49% in the SC IFX and IV IFX arms, respectively, *P* = .38) and serious TEAE at W30 (3.0% and 6.2% patients, *P* = .44) were similar between study arms. No patients receiving SC IFX had TEAEs leading to study drug discontinuation.^8^ In the LIBERTY clinical trial 72% and 61% in the CD study and 68% and 59% of patients in the UC study experienced at least 1 TEAEs in the SC IFX and placebo groups, respectively. Similar proportion of the SC IFX and placebo groups had TEAEs leading to study drug discontinuation.^11^ Injection site reaction, all nonserious, were reported by 16.7% of patients receiving SC IFX by Schreiber et al.^8^ Interestingly, local site pain generally decreased with repeated SC IFX administration. In the LIBERTY trials, injection site reactions during the maintenance phase were reported for 5.9% and 1.0% among the CD patients enrolled in the SC IFX and placebo groups, respectively; and for 3.4% and 2.9% of UC patients, respectively. Similarly, no cases were severe.^11^

Real-world studies confirmed that switching to SC IFX was well tolerated.^34^ In the long-term follow-up of the REMSWITCH study, 23% of the patients reported an adverse event after a median follow-up of 18 months. None was considered as severe adverse event, and none led to treatment discontinuation.^13^ Similarly, in the Liverpool cohort, there were no serious adverse reactions to treatment over the 12-month period and 3% had self-limiting skin injection reactions.^13^ In a recent meta-analysis including 15 studies and 1371 patients switched from IV to SC IFX, the pooled AE rate of 5.7% and SAE of 0.2%. The commonest AE was mild injection reactions (34/77 events, 44%).^20^

Interestingly, despite the higher serum drug concentrations with SC IFX compared to IV, the safety profile remained comparable. This finding is consistent with previous studies that found no correlation between IFX trough levels and the incidence of adverse events, such as infections or paradoxical reactions.^37^ Furthermore, in both clinical trials and real-world studies, dose intensifications were not associated with an increased occurrence of adverse events.^22^

## Acceptability of SC IFX and switch acceptance

The nationwide ACCEPT-2 study previously reported the preferences of SC rather than IV treatment by patients with IBD.^18^ The main factors reducing acceptability of IV therapy was to come to the hospital, the time spent at the infusion unit and missing a working day. Thus, SC IFX is an attractive option to limit this burden.^38^ Acceptability of SC IFX has been assessed using a 10 points-acceptability numerical scale (ANS) at different time points during the REMSWITCH trials.^13^^,^^14^ Patients’ acceptability was improved after switching from IV to SC IFX (mean ANS = 6.9 ± 1.6 vs. 8.6 ± 1.4; *P* < .0001) and did not diminish over time (8.8 ± 1.3 at 6 months and 8.8 ± 1.3 at 18 months; *P* < .001 for these 2 comparisons to baseline).^13^^,^^14^ However, some patients are reluctant to switch from IV to SC IFX. The experience from the Nancy IBD cohort, reported that 75.5% of the patients (*N* = 94 patients) treated with IFX accepted to switch. Median duration of treatment at inclusion was 7.0 years (3.0-11.0). While the main reasons for patient’s refusal for switching from IV to SC formulation were the potential impact on medical follow-up (39.1%) and fear of loss of efficacy (34.0%), a short duration of treatment was associated with a high switch acceptance rate (OR = 0.9 [0.8-0.9], *P* = .0002).

## Conclusions

Data from the pivotal clinical trial and real-world evidence show that SC IFX is a safe, well-accepted and effective option in patients with IBD. However, there are still data gaps to address to precise the position of SC IFX in some special situations.

## Supplementary Material

otag010_Supplementary_Data

## Data Availability

Data are available from the authors upon reasonable request.
