# Supplementary material for: Subcutaneous infliximab in inflammatory bowel disease: bridging the gap between theory and practice
Source: Crohns Colitis 360. 2026 Feb 5;8(1):otag010. doi: 10.1093/crocol/otag010 (PMC12958115; doi:10.1093/crocol/otag010)
Supplement: otag010_Supplementary_Data [file otag010_supplementary_data.docx]

**SUPPLEMENTARY MATERIAL**

**Supplementary Table 1**: List of relevant articles

|  | Population | n | Type of studies |
| --- | --- | --- | --- |
| 1. Hanauer et al. Lancet Lond Engl 2002;359:1541-1549. | Crohn’s disease | 573 patients | RCT |
| 1. Rutgeerts et al. N Engl J Med 2005;353:2462-2476. | Ulcerative colitis | 364 | RCT |
| 1. Sands et al. N Engl J Med 2004;350:876-885. | Fistulizing Crohn’s disease | 306 | RCT |
| 1. Laharie et al. Lancet Lond Engl 2012;380:1909-1915. | Severe ulcerative colitis | 115 | RCT |
| 1. Colombel et al. N Engl J Med 2010;362:1383-1395. | Crohn’s disease | 169 | RCT |
| 1. Kennedy et al. Lancet Gastroenterol Hepatol 2019;4:341-353. | Crohn’s disease | 955 | Cohort  Prospective |
| 1. Hemperly et al. Clin Pharmacokinet 2018;57:929-942. | Crohn’s disease  Ulcerative colitis | NA | Review |
| 1. Schreiber et al. Gastroenterology 2021;160:2340-2353. | Crohn’s disease  Ulcerative colitis | 131 | PK |
| 1. Kim et al. Innovative approaches to biologic development on the trail of IFX: biosimilars, value-added medicines, and biobetters. mAbs 2021;13:1868078. | NA | NA | Article |
| 1. Vuitton et al., Journal of Crohn’s and Colitis. 2024;18(Supplement_1):i1743-i1744; [ECCO 2024; Presentation number P960]. | Crohn’s disease  Ulcerative colitis | 131 | PK |
| 1. Hanauer et al. Gastroenterology 2024;-:1–15 | Crohn’s disease  Ulcerative colitis | 396 | RCT |
| 1. Kim et al, Gastroenterology, 2024 | Crohn’s disease  Ulcerative colitis | 485 | RWE |
| 1. Buisson et al. Clin Gastroenterol Hepatol Off Clin Pract J Am Gastroenterol Assoc August 2022:S1542-3565(22)00776-5 | Crohn’s disease  Ulcerative colitis | 184 | Observational study |
| 1. Buisson et al. Aliment Pharmacol Ther 2024;59:526-534. | Crohn’s disease  Ulcerative colitis | 128 | Observational study |
| 1. Huguet et al. Biomedicines 2022;10:2130. | Crohn’s disease  Ulcerative colitis | 30 | Prospective study |
| 1. Smith et al. J Crohns Colitis 2022;16:1436-1446. | Crohn’s disease  Ulcerative colitis | 181 | Cohort  Retrospective |
| 1. Remy et al. J Clin Med 2022;11:7296. | Crohn’s disease  Ulcerative colitis | 130 | Cohort Prospective |
| 1. Buisson et al. Inflamm Bowel Dis 2023;29:579-588. | Crohn’s disease  Ulcerative colitis | 1850 | RWE  Cohort |
| 1. Bothorel et al. Dig Liv DIs 2024 et mathieu et al. Clin gastroenterol Hepatol | Crohn’s disease  Ulcerative colitis | 360 | Retrospective cohort study |
| 1. Chetwood et al. J Crohns Colitis April 2024:jjae059. | Crohn’s disease Ulcerative colitis | 1371 | Systematic Review and Meta-analysis |
| 1. Qiu et al. J Gastroenterol 2017;52:535-554. | Crohn’s disease | 86 | Systematic Review and Meta-analysis |
| 1. Mathieu et al. Clin Gastroenterol 2025 | Crohn’s disease  Ulcerative colitis | 426 | Prospective cohort |
| 1. Wang et al. Clin Gastroenterol Hepatol. 2023 Nov;21(12):3188-3190 | Crohn’s disease  Ulcerative colitis | NA | PopPK simulation study |
| 1. Roblin et al. J Crohns Colitis November 2023:jjad188 | Crohn’s disease  Ulcerative colitis | 71 | Cross-sectional study |
| 1. Roblin et al. Aliment Pharmacol Ther. 2022 Jul;56(1):77-83. | Crohn’s disease | 20 | PK study |
| 1. Cerna et al. Crohn Colitis 360 | Cerna et al. Crohn Colitis 360 | 32 | RWE |
| 1. M. Andre et al. Poster P857, ECCO 2024 | Perianal Crohn’s Disease | 192 | Cohort |
| 1. Buisson et al.   Clin Gastroenterol Hepatol Off Clin Pract J Am Gastroenterol Assoc October 2023:S1542-3565(23)00766-8. | Crohn’s disease  Ulcerative colitis | 130 | Cohort  Post hoc |
| 1. Yarur et al   Journal of Crohn’s and Colitis. 2024;18(Supplement_1):i1930-i1931; [ECCO 2024; Presentation number P1074] | Crohn’s disease | 231 | RCT  Post hoc |
| 1. Caron et al. Aliment Pharmacol Ther. 2022;55:508–509 | Crohn’s disease  Ulcerative colitis | 4 | Cohort |
| 1. Husman J et al., Int J Colorectal Dis. 2024 | Crohn’s disease | 20 | Retrospective cohort study |
| 1. Vermeire et al. Gut. 2007; 56:1226-31 | Crohn’s disease | 174 | Cohort |
| 1. Schreiber et al. Inflamm Bowel Dis. 2025 Apr 30:izaf038. | Crohn’s disease (CD)  Ulcerative colitis (UC) | 192 CD  237 UC | RCT  Post hoc |
| 1. D'Haens and al.   Clin Drug Investig. 2023 Apr;43(4):277-288 | Crohn’s disease (CD)  Ulcerative colitis (UC) | 66 | RCT  Post hoc |
| 1. Gianolio et al. Journal of pediatric gastroenterology and nutrition, 77(2), 235-239. | Crohn’s disease  Ulcerative colitis  Peditaric | 7 | RWE |
| 1. Duquerois et al.   oral presentation JFHOD C.077 | Crohn’s disease  Ulcerative colitis  Pediatric | 21 | Cohort, retrospective |
| 1. Chetwood et al. J Crohns Colitis. 2024 Sep 3;18(9):1440-1449. doi: 10.1093/ecco-jcc/jjae059. PMID: 38656784. | Crohn’s disease  Ulcerative colitis | 1371 | A Systematic Review and Meta-analysis |
| 1. Bhattacharya et al. J Clin Gastroenterol. 2023 Jan 1 | Crohn’s disease | 341 | Cohort, retrospective |
| 1. Buisson et al.   Inflamm Bowel Dis 2013;19:2464-2467. | Crohn’s disease  Ulcerative colitis | 137 | Prospective cross-sectional study |

PK, pharmacokinetic; popPK, population pharmacokinetic; RCT, randomised controlled trial; RWE, real world evidence
